# Anatomical Structures as Graph Nodes: Representation Before Architecture in Paediatric Brain Tumour Localisation

**DOI:** 10.3390/diagnostics16213436

**Published:** 2026-10-22

**Authors:** Ayşe Keles

**Affiliations:** 1 School of Computer Science, University of Galway, H91 TK33 Galway, Ireland; ayse.keles@universityofgalway.ie; 2 Department of Computer Engineering, Faculty of Engineering and Natural Sciences, Ankara Medipol University, 06570 Ankara, Turkey

**Keywords:** paediatric brain tumour, graph neural network, graph attention network, representation learning, anatomical prior, magnetic resonance imaging, tumour localisation, small-cohort deep learning

## Abstract

**Background/Objectives:** Paediatric brain tumour cohorts are small, and deep networks trained directly on volumetric MRI often perform poorly on them. The usual response is a larger architecture. The contribution of the anatomical representation itself has not been isolated as an experimental factor, so it is not known how much of the difficulty is architectural and how much is representational. This study addresses that gap, using supratentorial/infratentorial tumour localisation as a task defined entirely by anatomical geometry. **Methods:** In 108 paediatric patients with multi-sequence MRI and manual annotation of the tumour and eight anatomical structures, the task, the cohort and the subject-grouped five-fold cross-validation assignment were held fixed while the representation of the input was varied: 3D convolutional networks on raw volumes, the same networks given coordinate and craniocaudal-profile priors, a geometric rule with no fitted parameters, and a patient-level graph whose nodes are anatomical structures, read out by logistic regression or by a graph attention network. All anatomy downstream of the landmarks was predicted out of fold. Comparisons were patient-paired, with bootstrap intervals and Holm-corrected McNemar tests. **Results:** Balanced accuracy rose from 0.522 on raw voxels to 0.652 with explicit spatial priors, 0.681 for the parameter-free geometric rule, 0.775 for a five-feature linear readout of the graph and 0.838 for the graph attention network; the same decision layer given manual masks reached 0.918. Only the graph attention network exceeded the strongest convolutional baseline significantly (+0.187, Holm-adjusted *p* = 0.009). Automated segmentation was associated with a 0.143 gap, driven by spatial mislocalisation rather than by missed tumours. **Conclusions:** For this geometrically defined task and cohort size, representing anatomical structure explicitly was more effective than learning the same geometry from voxels.

## 1. Introduction

Brain and other central nervous system tumours are the leading cause of cancer-related death among children and adolescents in recent United States population-based data [1]. Among the first questions asked of a new scan is where the tumour sits relative to the tentorium cerebelli: the supratentorial/infratentorial distinction informs the differential diagnosis, contributes to operative planning, and is associated with complications such as obstructive hydrocephalus. It is, at root, a question of geometry.

Reported performance in paediatric brain tumour AI studies nonetheless varies substantially with task definition, cohort size, imaging protocol and evaluation design, and relatively little work has isolated the contribution of an explicit anatomical representation. The largest published paediatric posterior-fossa series contain a few hundred patients—617 [2], 527 [3], 288 [4], 158 [5]—and single-centre cohorts are typically far smaller. At this scale, a 3D convolutional network trained end-to-end on multi-sequence volumes has on the order of 10^7^ input dimensions and 10^2^ training examples. The usual response to poor performance is architectural: a deeper backbone, a transformer, more augmentation, a pretrained encoder.

This study takes the opposite position. The task, the cohort and the cross-validation folds are held fixed, and only how the input is represented is varied—from raw voxels, through increasingly explicit geometric encodings, to a graph whose nodes are named anatomical structures. If performance rises along that axis while architectural capacity does not, the bottleneck is representational.

**Figure 1:**
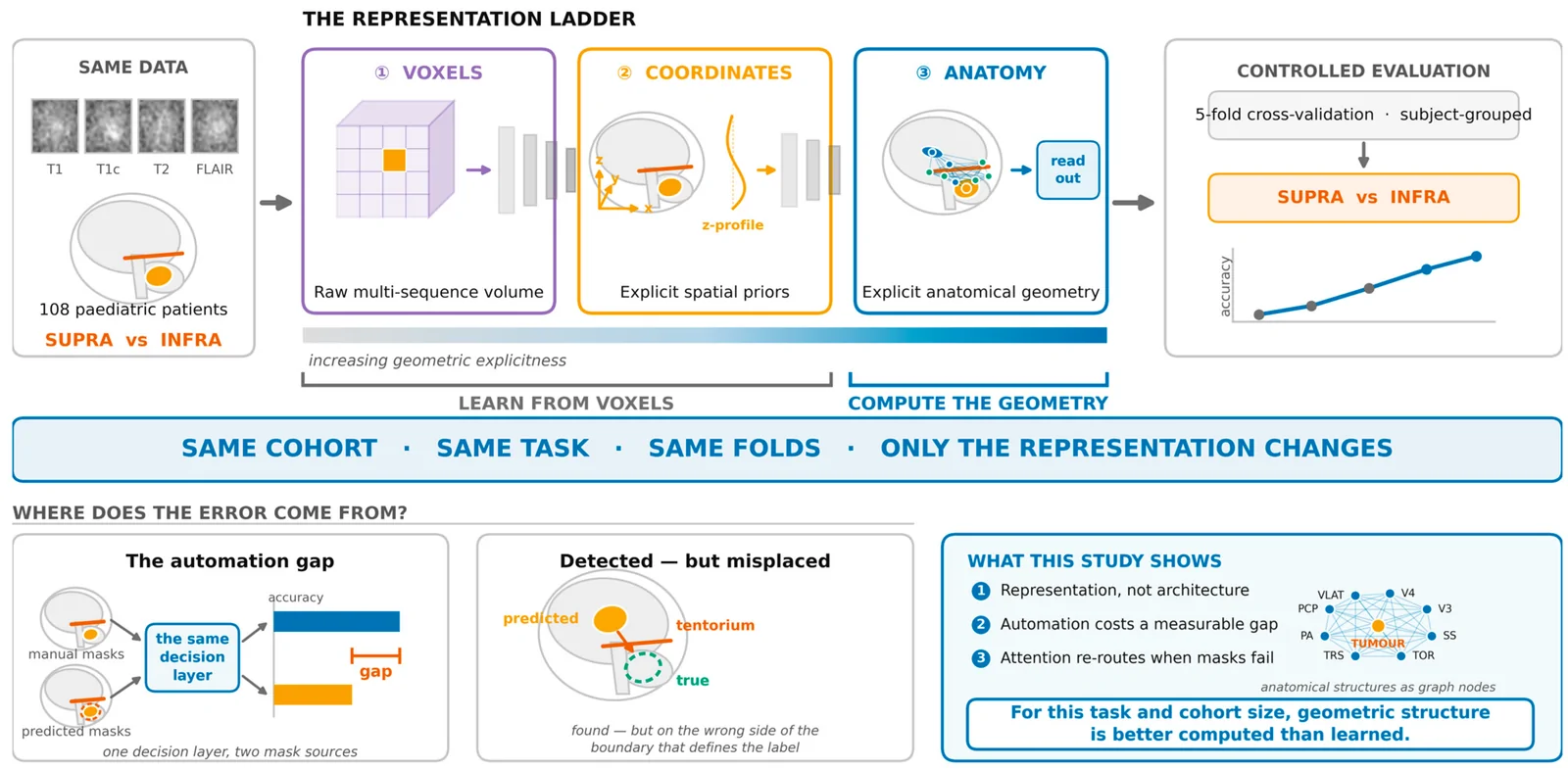
**Study design.** One paediatric cohort, one classification task and one subject-grouped five-fold cross-validation assignment are held fixed while the representation of the MRI is varied along three rungs: the raw four-sequence volume; the volume with explicit coordinate and craniocaudal-profile priors; and an anatomical landmark graph in which nine segmented structures become nodes carrying a normalised centroid, log volume and presence flag. The first two rungs leave geometry to be learned by a 3D CNN; the third computes it from masks. Numerical values are reported in Section 3 and are deliberately omitted here. The lower panels show the two error analyses the comparison supports and a summary of what follows from them.

Figure 1 states the design. One paediatric cohort, one classification task and one fold assignment are held fixed; only the representation of the MRI changes, from raw voxels through explicit spatial priors to an anatomical landmark graph.

Research on paediatric posterior fossa tumours is dominated by convolutional and radiomic pipelines. Ref. [2] trained a modified ResNeXt-50 multitask classifier on 617 children across five institutions; Ref. [4] compared automated and manually optimised radiomics pipelines on 288 patients; Ref. [5] fused a pretrained ResNet-50 with tabular age data on 158 scans; and Ref. [3] built a sequential classifier over Image Biomarker Standardisation Initiative (IBSI) features on a multinational cohort of 527. Ref. [6] systematically reviewed 25 such algorithms. Closest to the present work, Refs. [7,8] trained two-dimensional deep networks with age fusion on CBTN subjects. Bianchessi et al. (181 subjects) report a Matthews correlation coefficient of 0.70 on apparent diffusion coefficient maps and 0.57 on T2-weighted images for a five-class task, and Tampu et al. (178 subjects) report 0.77 ± 0.14 and 0.58 ± 0.11 for a three-class task restricted to the infratentorial compartment. In both, the quantity being predicted is tumour type—low-grade astrocytoma, ependymoma, medulloblastoma. Location is not absent from that work: Bianchessi et al.’s five-class label compounds type with compartment, so supratentorial and infratentorial variants of a type form separate classes. But compartment is never the target on its own, and no accuracy for localisation as such is reported, so those figures are not a baseline for the task studied here. None of these studies uses a graph representation, and none treats the representation of anatomical geometry as the experimental variable.

The prevailing response to limited accuracy in this domain has been architectural. Recent work continues to refine backbone design and attention mechanisms for brain tumour MRI: Ref. [9], for instance, augment EfficientNetV2 with global and efficient channel attention and report 99.76% accuracy on a four-class brain tumour MRI benchmark, among the strongest results published on that collection. Such results establish that architectural refinement is close to saturating what these benchmarks can measure, which is part of the motivation for asking whether the remaining difficulty in small clinical cohorts lies elsewhere. A caution applies when reading accuracies of this magnitude alongside the ones reported here: figures in the 95–100% range are typically obtained on public slice-level collections (BraTS derivatives, Kaggle repositories) in which slices from one patient can fall on both sides of the train/test split, and in which the task is tumour type on a curated, largely adult or mixed-age population. The numbers in this paper are patient-level, subject-grouped and out-of-fold on a paediatric cohort of 108, and the two are not comparable.

Where graphs do appear in brain tumour imaging, they are constructed in one of two ways. In pixel or superpixel graphs, Ref. [10] apply a Prewitt edge operator and designate pixels with intensity above a fixed threshold as nodes, while Ref. [11] use a precomputed adjacency matrix over pixel neighbourhoods. In CNN feature graphs, Refs. [12,13,14,15,16] extract features with a convolutional or transformer backbone and treat those features as nodes. In both families the graph is a reparametrisation of the image grid or of learned features rather than of named anatomical structures. Graphs over anatomical landmarks appear elsewhere in medical imaging, in landmark detection for instance, but no prior use of them as the patient representation for a brain tumour classification task was identified.

Two properties motivate anatomical structures as graph nodes for this task. First, the quantity that defines the label is relational: the compartment of a lesion is its position relative to the tentorium, and the tentorium is itself defined by a set of named bony and venous landmarks. A representation whose primitives are those landmarks encodes that relation directly, whereas a voxel grid encodes it only implicitly and must rediscover it from data. Second, the representation is small. Nine structures described by a normalised centroid, a log volume, a presence flag and an identity code reduce the input from roughly 10^7^ dimensions to 126, which is what makes a model with fewer than five thousand parameters viable on a hundred patients. The anatomical prior supplies what would otherwise have to come from training examples that do not exist.

The limitations of voxel-based models in this task are related to the need to identify the tumour’s position relative to the tentorium. In 3D CNNs, global average pooling summarises spatial features and may reduce information about the tumour’s exact location. As a result, similar tumours above and below the tentorium may produce similar feature representations. U-Net architectures preserve spatial information through skip connections, but a classification head may still lose positional information during pooling. Transformers can encode absolute position using positional embeddings; however, their greater modelling capacity may not provide an advantage when the training cohort is small. These considerations motivate the comparison of raw-voxel and coordinate-channel models with a craniocaudal-profile approach, which preserves positional information along the superior–inferior axis. The results in Section 3.1 show how much this approach improves performance.

Graphs whose nodes are real biological entities are, by contrast, well established in pathology. Ref. [17] derive prognostic features from whole-slide tumour microenvironment graphs; Ref. [18] model spatial protein profiles as local cellular subgraphs; and Ref. [19] model tumour–immune interactions in non-small-cell lung cancer (NSCLC). These demonstrate that the graph paradigm pays off precisely when nodes carry biological meaning—but they operate on tissue rather than on radiology.

To the best of my knowledge, no published study has askedwhether the finding survives contact with an automated pipeline, because every mask in it is drawn by hand. The present paper differs in four respects: all anatomy downstream of the landmarks is predicted out of fold rather than annotated; the representation is a patient-level graph rather than a set of image channels; the task is restricted to two classes so that a like-for-like comparison against voxel models is possible at this cohort size; and the analysis extends to what happens to each representation when the upstream segmentation fails. The geometric construction is specified in full in Section 3.2.

Existing paediatric neuro-oncology studies ask mainly whether a model can classify the tumour. The question posed here is methodological: how much of a geometrically defined task becomes solvable when the anatomy that defines it is made explicit, and where does the remaining error come from? Previous studies have applied deep learning to paediatric brain tumour classification and localisation, but the effect of the anatomical representation has not been isolated as an experimental factor. In particular, a patient-level graph built from explicitly annotated anatomical structures does not appear to have been used as a controlled alternative to voxel-based learning, with cohort, task and subject-level folds held fixed.

The main contributions are:

1. **A controlled comparison of representations.** The same cohort is evaluated under the same patient-level fold assignment while the representation of anatomical information is changed progressively: from 3D CNNs on raw MRI volumes, to CNNs given coordinate and z-profile information, to anatomical landmark graphs. A decision rule with no learned parameters is included as a reference point (Section 3.1 and Section 3.2).
2. **A direct measurement of the automation gap.** The same decision layer is compared when it receives manually annotated masks and when it receives automatically predicted ones, isolating the difference introduced by replacing manual anatomy with automated segmentation (Section 3.4).
3. **An analysis of how segmentation errors affect localisation.** The dominant failure was spatial misplacement of the tumour prediction; complete absence of a prediction was rare. The error analysis shows this mislocalisation to be systematic rather than random (Section 3.4).
4. **A localisation of the mechanism behind the graph model’s robustness.** The hypothesis that the model re-routes attention away from an unreliable tumour node is tested separately at the two stages where attention acts. No such effect was found at the readout, whereas attention in the first message-passing layer changed clearly. This identifies the stage at which the behaviour arises, and shows why examining the readout alone would have led to the opposite conclusion (Section 3.5 and Section 3.6).

These four contributions map onto Section 3 in order: the controlled comparison of representations in Section 3.1, Section 3.2 and Section 3.3, the automation gap in Section 3.4, the analysis of how segmentation errors propagate in Section 3.4, and the localisation of the mechanism in Section 3.5 and Section 3.6. Section 5 returns to them in the same order.

This paper does not argue that CNNs are unsuitable for medical imaging. It asks a narrower question: when the target is defined by anatomical geometry and the available cohort is small, should that geometry be learned from voxels or represented explicitly? The results reported here indicate that, for this task, making the geometry explicit provides a substantial advantage, and they quantify the margin.

## 2. Materials and Methods

### 2.1. Study Cohort and Annotation

The study cohort consisted of 108 paediatric patients with multi-sequence brain MRI, including T1, T2, FLAIR and T1ce sequences, and was assembled from two sources. Seventeen patients were obtained from the Children’s Brain Tumor Network (CBTN [20]). The remaining 91 patients came from a retrospective clinical archive compiled by the collaborating neurosurgeon in the course of her surgical practice. Because she operated at more than one centre over the period covered, the archive is not confined to a single hospital; it is held by its custodian, who is currently affiliated with the Interventional MR Clinical Research and Development Institute (GİMRE), İbni Sina Hospital, Ankara University, Ankara, Türkiye. Retrospective use of this archive was approved by the ethics committee named in the back matter, and all imaging was de-identified before analysis. Only patients meeting the predefined inclusion criteria for the supratentorial/infratentorial localisation task were included in the analysis. Patients were excluded if their annotations indicated both supratentorial and infratentorial involvement, or if the lesion was outside the anatomical scope of the study; the latter included extra-cranial lesions, such as spinal and vertebral masses, for which a supratentorial/infratentorial label is not anatomically defined. These criteria were established before model development and were applied independently of the modelling results. The resulting class balance was 57 supratentorial and 51 infratentorial patients, so chance performance is 0.500 throughout.

The two sources are not balanced with respect to class: 44% of local cases are infratentorial versus 65% of external cases. Because the external cases also differ in acquisition protocol, source is a potential confounder, and Section Limitations and Future Directions returns to this point.

**Figure 2:**
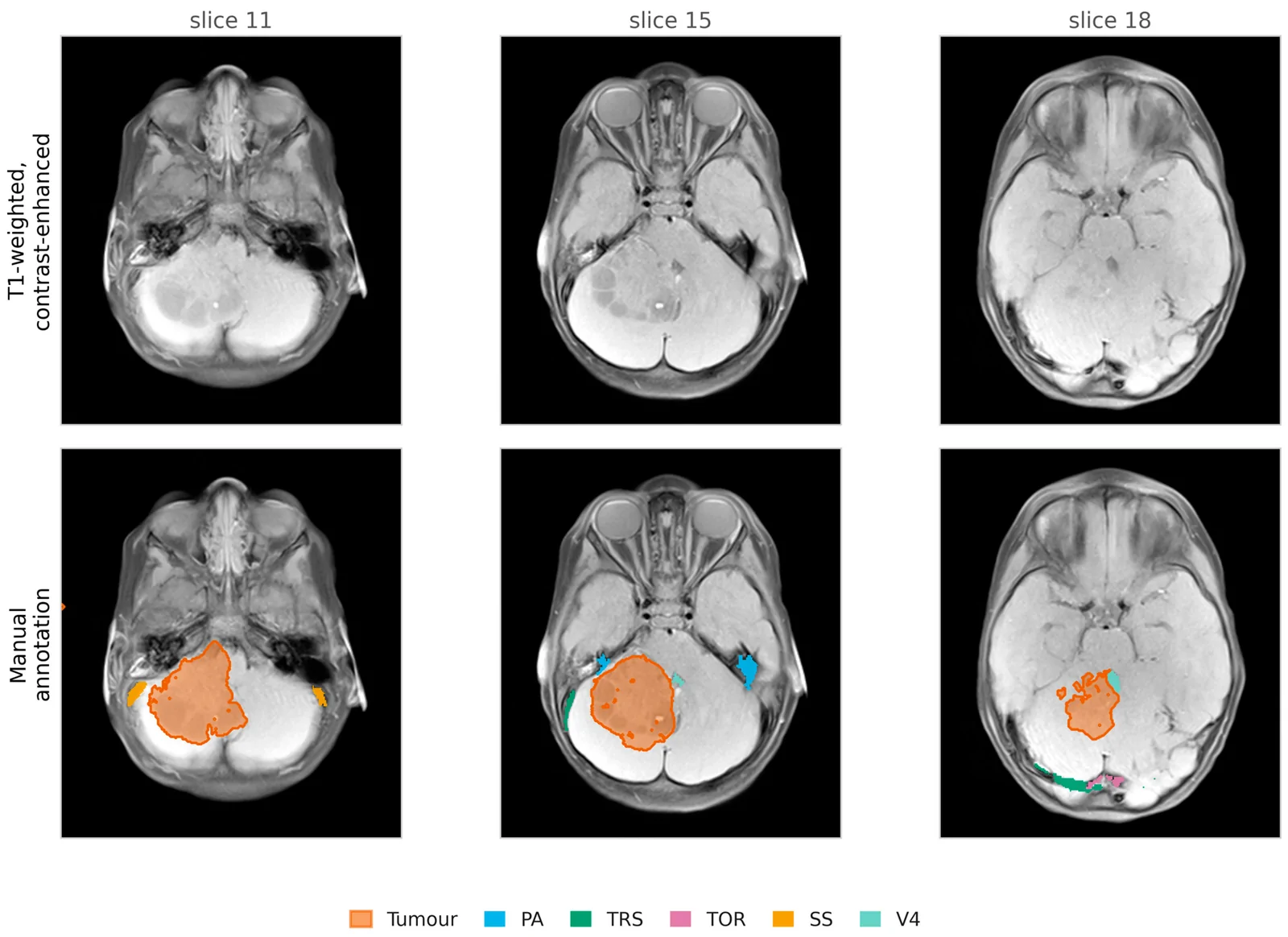
**Manual annotation of the tumour and the anatomical landmarks.** Three axial slices of an infratentorial tumour case. Top row: contrast-enhanced T1-weighted images. Bottom row: the same slices with the manual annotation overlaid. Abbreviations: PA, petrous apex; TRS, transverse sinus; TOR, torcular Herophili; SS, sigmoid sinus; V4, fourth ventricle.

All included patients had manual annotations of the tumour and of the relevant anatomical structures. The stage-1 segmentation task used nine target classes: TUMOUR (all tumour sub-labels merged), five tentorial landmarks—posterior clinoid process (PCP), petrous apex (PA), transverse sinus (TRS), torcular Herophili (TOR) and sigmoid sinus (SS)—and three ventricular structures: third ventricle (V3), fourth ventricle (V4) and lateral ventricles (VLAT). Tumour sub-compartments (solid, core, oedema, necrosis, haemorrhage, contrast-enhancing) were annotated separately and merged for this task. Structures whose annotation coverage was insufficient were not included as target classes. Ethical approval and data-use agreements are given in the back matter. Figure 2 shows an example. The panels pair three axial slices of a contrast-enhanced T1-weighted volume with the corresponding manual annotation, in an infratentorial case in which the tumour sits below the tentorial attachment and the annotated landmarks bracket it.

The supratentorial/infratentorial reference label was assigned separately from the voxel annotation. For every patient the collaborating neurosurgeon reviewed the diagnostic images and recorded the compartment in a structured clinical review table, alongside laterality, mass effect and the working diagnosis, judging the position of the bulk of the lesion relative to the tentorium cerebelli from the images themselves. That table, and not the drawn masks, is the source of the labels used throughout this study. The label was therefore not derived from the annotations that later supply the model features, and the two are independent by construction; the same review also fixed the inclusion criteria described above. The annotations themselves were produced by the clinical collaborators named in the Acknowledgments, and each case was read once. No second reader or adjudication step was available, so inter-rater agreement could not be quantified (Section Limitations and Future Directions).

### 2.2. Image Preprocessing

Volumes are converted to canonical RAS (right–anterior–superior) orientation, cropped to a brain bounding box and resampled to 256 × 256 × 32. Orientation matters here: the cohort contains three header orientations (LPS 112, LAI 22, LAS 7) and in LAI cases the third axis is inferior-directed—that is, 15.6% of patients were craniocaudally inverted in the raw voxel order, along exactly the axis that carries the supratentorial/infratentorial label. All results in this paper use the canonicalised cache. Resampling to a 32-slice craniocaudal grid is lossy, and because the label is defined along exactly that axis, the loss is not incidental. Its cost is therefore measured rather than assumed: Section 3.2 compares the tentorial rule computed in the original millimetre space with the same rule computed in the resampled cache, and finds a loss of 0.019 balanced accuracy for the uncalibrated rule and none at all once a single threshold is fitted. The resampling discards tentorial tilt, but not the information the task depends on.

### 2.3. Stage-1 Anatomical Segmentation

The architecture is a ViT-Mamba3D encoder–decoder with 16 base channels, taking the four co-registered sequences as input channels and producing ten output classes: background, tumour, the five tentorial landmarks and the three ventricular structures. The model is trained within each fold. For each fold, the model was trained on the fold-specific training patients and the 16 bitentorial patients excluded from the classification task. These additional patients were used only for segmentation and were absent from all test folds. Data augmentation comprised translations (±4 in-plane voxels and ±2 craniocaudal slices) and intensity jitter. Flips and 90° rotations were excluded to preserve anatomical orientation and spatial relationships required by the downstream task.

The loss function combined Focal Tversky (α = 0.7, β = 0.3, γ = 1.3), soft Dice and cross-entropy losses with weights of 0.5, 0.4 and 0.1, respectively. Missing annotations were handled through probability marginalisation, preventing unannotated classes from being penalised as false positives. To improve the learning of small structures, the five landmarks were dilated by class-specific margins: PCP, 3.0 mm; TOR, 2.0 mm; and PA, TRS and SS, 1.5 mm.

The model was optimised using AdamW (learning rate 3 × 10^−4^; weight decay 10^−5^), cosine annealing, gradient accumulation of four and bfloat16 mixed precision. Training continued for up to 60 epochs, with early stopping after 15 epochs without improvement. The checkpoint with the highest mean validation Dice was selected using a patient-grouped, class- and cohort-stratified validation split comprising 15% of the fold’s training patients. Test-fold data were excluded from training and model selection.

These settings were inherited from the pre-existing segmentation pipeline and selected based on validation Dice and anatomical validity rather than classification performance. Landmark dilation was the only modification explicitly motivated by the downstream task. Its geometric impact was assessed separately: a 2 mm landmark radius changed the fitted surface normal by a mean of 0.6° and a maximum of 2.9°, without changing the decision sign. Out-of-fold segmentation performance for each structure is reported in Section 3.4.

### 2.4. The Anatomical Landmark Graph

Each patient is represented by an undirected, fully connected graph over the nine predicted structures. Every node carries a fourteen-dimensional feature vector: the structure’s centroid expressed in a landmark reference frame (3 dimensions), the logarithm of its voxel count (1), a presence flag set to one when the structure was predicted and zero otherwise (1), and a one-hot encoding of node identity (9).

The reference frame is derived from the available tentorial landmarks. Let *L* ⊆ {PCP, PA, TRS, TOR, SS} be the subset of those five landmarks predicted in a given patient, and c_l_ the voxel centroid of landmark *l*. The frame origin is the mean of the available landmark centroids,

**μ** = (1/|*L*|) Σ*_l_*∈ *L* **c**_l_, (1)

and the scale is their root-mean-square spread about that origin,

*s* = [(1/|*L*|) Σ*_l_*∈ *L* ‖**c**_l_ − **μ**‖^2^]^(1/2). (2)

Every node centroid c_i_ is then expressed in that frame as

**c̃**_i_ = (**c**_i_ − **μ**)/max(*s*, ε), (3)

with ε a small constant guarding against a degenerate landmark configuration. All coordinates are measured in the resampled voxel space described in Section 2.2. Equations (1)–(3) define a translation-and-scale normalisation only; no rotation is estimated because the volumes are already in canonical RAS orientation. When |*L*| < 3 the frame is not identifiable and a fixed frame—the volume centre and half the volume diagonal—is substituted; on predicted masks this never occurred (105 of 108 patients had all five landmarks and 3 had four), and on manual masks it occurred once. Absent nodes are zeroed and masked out of attention as keys.

### 2.5. Model and Input Configurations

The five configurations compared in this study are listed in Table 1. They share the cohort, the label and the fold assignment, and differ only in what reaches the decision layer.

The three CNN variants are: scratch3d (raw four-channel volume), scratch3d_coord (with explicit coordinate channels) and scratch3d_zprofile (with a craniocaudal profile encoding). They form the intermediate rungs of the ladder: each supplies the network with progressively more geometric information while leaving the architecture family unchanged.

### 2.6. Graph Attention Network

Each node carries the fourteen-dimensional feature vector defined in Section 2.4. The graph readout is a graph attention network (GAT) with two message-passing layers, two attention heads per layer and a hidden width of 16. Attention is computed over the fully connected graph, and the attention logit of an ordered pair of nodes carries a learned distance penalty, *att_ij_* − softplus(*γ*)·*d_ij_*, in which *d_ij_* is the Euclidean distance between the two node centroids in the normalised frame and *γ* is a single learned scalar. The softplus keeps the penalty non-negative, so that spatially close structures form stronger edges while the strength of that preference is learned rather than imposed. Nodes absent in a given patient are masked out as attention keys. The graph-level vector is formed by attention pooling against a learned query, and one linear layer maps it to the two classes.

The model was trained with dropout of 0.3, using AdamW with a learning rate of 3 × 10^−3^ and weight decay of 10^−2^. Training was performed for 200 epochs using the full training set in each fold and a class-weighted cross-entropy loss. The network contains 4772 trainable parameters, and training took approximately 4 s per seed on an RTX 4090.

No early stopping was used. This avoided selecting a model checkpoint based on performance on the test fold, which could introduce information leakage. All hyperparameters were fixed before evaluating the results and were not tuned against the test data.

### 2.7. Evaluation Protocol

- Five-fold cross-validation with StratifiedGroupKFold, grouped by subject (repeat sessions of the same child form one group) and stratified by class × cohort, seed 0.
- No stage leakage. Predicted masks are out-of-fold: a patient in the stage-3 test fold was also held out of stage-1 training.
- Fold identity verified. The same fold assignment was used for all CNN and graph-based models. The fold identities were verified for all 108 patients, ensuring that model comparisons were performed on the same test cases and were therefore paired.
- Environment fixed. The software environment was fixed because StratifiedGroupKFold can produce different fold assignments across scikit-learn versions. Analyses were run under Python 3.12.8 with NumPy 2.3.5, SciPy 1.15.3, scikit-learn 1.8.0, PyTorch 2.11.0+cu128 and NiBabel 5.3.3 on an NVIDIA RTX 4090 (NVIDIA Corporation, Santa Clara, CA, USA), and the exact fold assignment used in this study is treated as part of the evaluation protocol.
- Metrics. Balanced accuracy (classes 57/51) and area under the receiver operating characteristic curve (AUC); 95% confidence intervals by bootstrap over patients (5000 resamples); paired comparisons by McNemar’s exact test.
- Ensembles. For the graph-based model, results are reported using a pre-specified ensemble of ten random seeds. Sensitivity to the number of seeds (m = 1–30) and matched-compute comparisons are reported in Section 3.5 and Section 3.3, respectively.

## 3. Results

### 3.1. Explicit Spatial Information Improves Localisation

**Figure 3:**
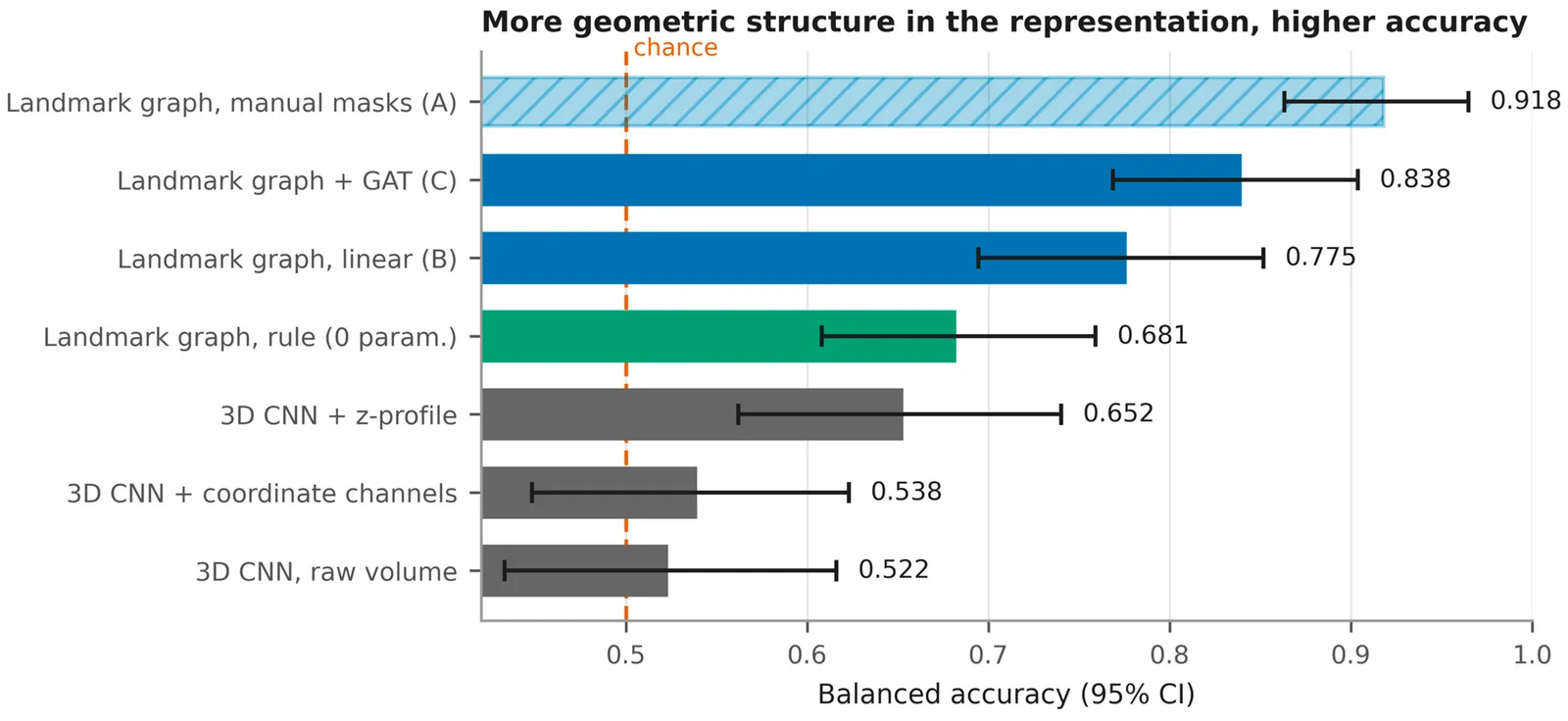
**The representation ladder.** Balanced accuracy for seven decision procedures over the same 108 patients under the same five-fold cross-validation assignment, with bootstrap 95% confidence intervals (5000 resamples). Grey: 3D CNN variants, each a ten-seed ensemble on the same fold partition. Green: the geometric rule with no fitted parameters. Blue: anatomical landmark graph configurations. Hatched: the manual-mask reference. Dashed vertical line: chance (0.500).

Balanced accuracy increased across the representation settings (Figure 3). On raw voxels a 3D CNN reached 0.522; adding coordinate channels raised this to 0.538, and adding a craniocaudal profile raised it to 0.652. A tentorial rule with no fitted parameters reached 0.681, a five-feature logistic regression on the landmark graph 0.775 (configuration B), and the GAT 0.838 (configuration C). The same decision layer supplied with manual masks reached 0.918 (configuration A). All three convolutional rungs use one protocol—ten random seeds combined by majority vote over the identical fold partition—so the comparison between them is like-for-like.

The ladder contains two complementary comparisons. The first three rungs keep the CNN architecture fixed and progressively add spatial information to the input; this isolates the effect of making geometry explicit to a convolutional network. The remaining rungs keep the anatomical information available to the decision fixed, and vary how that information is used: an unfitted geometric rule, a fitted linear readout over the tumour node, a GAT over all nine nodes, and finally the same linear readout supplied with manual rather than predicted masks. The comparison is not perfectly controlled across the second group—the tentorial rule uses the landmark point clouds and the tumour centroid directly, not the nine-node graph—and it is presented as a progression of decision procedures rather than as a single-variable experiment. The last rung is a reference, not a ceiling: it bounds this pipeline under perfect segmentation, not the task.

The largest single step in the second group, 0.681 → 0.775, is not the arrival of a neural network. It is the introduction of a learned decision boundary. The ladder design starts an explicitly computed geometric rule from the earlier, unpublished work, which established on the manually annotated version of this cohort assigns the compartment accurately with no fitted parameters; the comparison against learned models, and the case where every mask downstream of the landmarks is predicted out of fold, are introduced here.

Two intermediate results are worth naming. Adding coordinate channels to the CNN helped little (0.522 → 0.538), whereas adding a craniocaudal profile helped substantially (→ 0.652). This suggests that the difficulty lies less in the absence of position information than in its loss through pooling; the graph representation avoids that loss because position is supplied directly as a node feature. Separately, more nodes did not help a linear model: configuration B′ over all nine nodes reached 0.750 (95% CI 0.666–0.831), below configuration B’s 0.775 with one node. At this cohort size each additional static feature costs more than it returns.

### 3.2. Simple Geometry Explains Much of the Task

Before asking what a model contributes, it is worth asking what the anatomy alone gives. The five tentorial landmarks lie along the tentorial attachment, so a surface fitted through them approximates the tentorium itself, and the sign of the tumour centroid’s offset from that surface is a complete decision procedure with no fitted parameters. That surface is not devised here: it is the anisotropy-aware weighted total-least-squares fit of the earlier work described in Section 1, used unchanged. Landmark voxels enter as point clouds with equal weight per landmark; the measurement covariance is diagonal and set to half the voxel size along each axis, so that the plane normal is the generalised-eigenvector solution of the weighted scatter matrix with the smallest eigenvalue and the plane passes through the weighted landmark centroid. The normal is oriented anatomically so that the lateral ventricles—supratentorial content—fall on its positive side. The contribution here is to apply it to predicted, out-of-fold masks (Table 2).

A procedure with no fitted parameters reached 0.777 on manual masks, and a single fitted threshold on the same signed distance reached 0.874. A single coordinate, the craniocaudal position of the tumour centre of mass, reached 0.856. At the level of the anatomy itself this task is therefore close to a sign test. This means the information that defines the label is present and low-dimensional; a 3D CNN trained on the same preprocessed volumes reached 0.614 averaged over ten seeds and did not recover it. This difference is the clearest indication in this study that the limitation is representational.

The manual-mask figures are oracle quantities and must not be compared with end-to-end results. The realistic counterpart of the single *z* coordinate, computed from predicted masks, is 0.759—below the five-feature linear readout (0.775) and well below the graph model (0.838). Comparing a manual-mask single feature against a predicted-mask model would set an upper bound against an end-to-end result, and that comparison is avoided here.

Although the anatomical boundary is not horizontal, the craniocaudal tumour coordinate captures most of the class separation in this cohort. The tentorium is a tent rather than a plane: the landmark ring departs from the fitted plane by a median peak-to-trough height of 19.7 mm, measured on the manual annotations in this cohort, and the fits performed here are consistent with a non-axial surface, with a median tilt of 7.1° off axial in the original millimetre space. A surface that is planar in the mean is therefore biased towards assigning borderline cases to the supratentorial side, and the sign-only rule is correspondingly asymmetric: on predicted masks it recovered 53 of 57 supratentorial cases but only 22 of 51 infratentorial ones. This is not an artefact of the preprocessing used here, because it persists when the same fit is computed in the original millimetre space (32 of 51 infratentorial cases were correctly classified there); one fitted threshold removes most of it, raising infratentorial recall to 40 of 51.

The rule also makes the cost of preprocessing measurable. All configurations operate on volumes resampled to 256 × 256 × 32, which compresses the craniocaudal axis far more than the in-plane axes and flattens the fitted tentorial normal from a median 7.1° off axial in the original millimetre space to roughly 1° in the resampled grid. That flattening costs little: on manual masks the sign-only rule loses 0.019 (0.796 in millimetre space versus 0.777 resampled) and the calibrated rule loses nothing (0.874 in both). The resampling discards tentorial tilt but not the information the task depends on.

Finally, the rule degrades under automation, falling from 0.777 to 0.681 without a fitted threshold and from 0.874 to 0.787 with one. The calibrated rule on predicted masks is not distinguishable from the five-feature graph readout (0.787 versus 0.775, McNemar *p* = 0.093) but remains significantly below the GAT (*p* = 0.0025). Hand-written geometry and a learned readout agree about what the task is; they differ in what happens when the geometry is measured imperfectly, which Section 3.5 examines directly.

### 3.3. Paired Comparisons Between Representations

All models were evaluated on the same fixed fold assignment, verified patient-by-patient for all 108 patients, so every comparison below is patient-paired and McNemar’s test applies. Each CNN baseline is a ten-seed ensemble on that partition, matching the graph model’s pre-specified ensemble size (Table 3).

The GAT exceeded the strongest CNN baseline by 0.187 balanced accuracy. The linear single-node readout did not (*p* = 0.073), and the graph model did not significantly exceed the linear readout (*p* = 0.092). All three comparisons are reported rather than the favourable one alone.

These three tests address one question—whether the graph representation improves on the strongest available alternative—and are corrected for multiplicity by the Holm–Bonferroni procedure. The comparisons against the two weaker CNN variants are descriptive rungs of the ladder rather than competing claims and are not included in the family. Adjusted values are: GAT_10_ versus the CNN ensemble, p_adj = 0.009; linear readout versus the CNN ensemble, p_adj = 0.145; GAT_10_ versus the linear readout, p_adj = 0.145. The single significant result survives correction and the two negative results are unchanged. The strongest CNN baseline was itself sensitive to random initialisation, with single-seed balanced accuracy ranging from 0.533 to 0.678 on a fixed partition, which is why every baseline is reported as a ten-seed ensemble. Because balanced accuracy averages the two class-wise rates, they are reported separately in Table 4.

Under manual annotation, the two classes are recovered almost equally. Under predicted masks, every configuration loses more on the infratentorial class, and the graph model’s gain over the linear readout is carried almost entirely by the supratentorial side rather than by the side that is failing. This asymmetry is not explained by differential upstream failure, since stage-1 misplaces the tumour at a similar rate in both classes (17 of 57 supratentorial cases, 17 of 51 infratentorial cases; median Dice scores of 0.562 and 0.559). It is more consistent with the decision boundary sitting slightly too high, in the same direction and plausibly for the same reason as the planar rule’s bias in Section 3.2. Threshold calibration would correct the symptom; the curvature of the boundary is the cause. The uncalibrated operating point, balanced accuracy at a 0.5 threshold, is reported throughout. Configuration A does not read the label from the annotation: it applies an out-of-fold linear classifier to five numbers derived from the manually annotated tumour node, so the compartment must still be inferred from geometry. Its nine errors were not distributed randomly. Expressed as the perpendicular offset of the tumour centre of mass from the plane of the tentorial landmarks, supratentorial patients had a median offset of +8.6 voxels (IQR +4.7 to +11.1) and infratentorial patients had a median offset of −0.7 voxels (IQR −1.5 to +1.1), so the two classes overlap in a band around the tentorial level. All nine errors fell inside that band, with offsets between −1.7 and +7.1 voxels, and none of the 21 patients lying outside it was misclassified. The misclassified tumours were also smaller than average, with a median volume below a third of the cohort median. Fitting the same five features to the whole cohort without cross-validation did not remove these errors (balanced accuracy 0.909, ten patients misclassified).

### 3.4. The Automation Gap and Its Failure Mode

The same decision layer reached 0.918 on manual masks and 0.775 on predicted masks. Automated segmentation is therefore associated with a gap of 0.143 balanced accuracy in this pipeline. This is stated as an association: the two configurations differ only in the source of the masks, but the gap has not been decomposed into contributing error modes.

Segmentation quality varied by an order of magnitude across the nine structures, and the node on which the decision rests is among the least reliable positionally (Table 5). The lateral ventricles are recovered well; the tumour node has the largest median centroid error of any structure except the sinuses.

**Figure 4:**
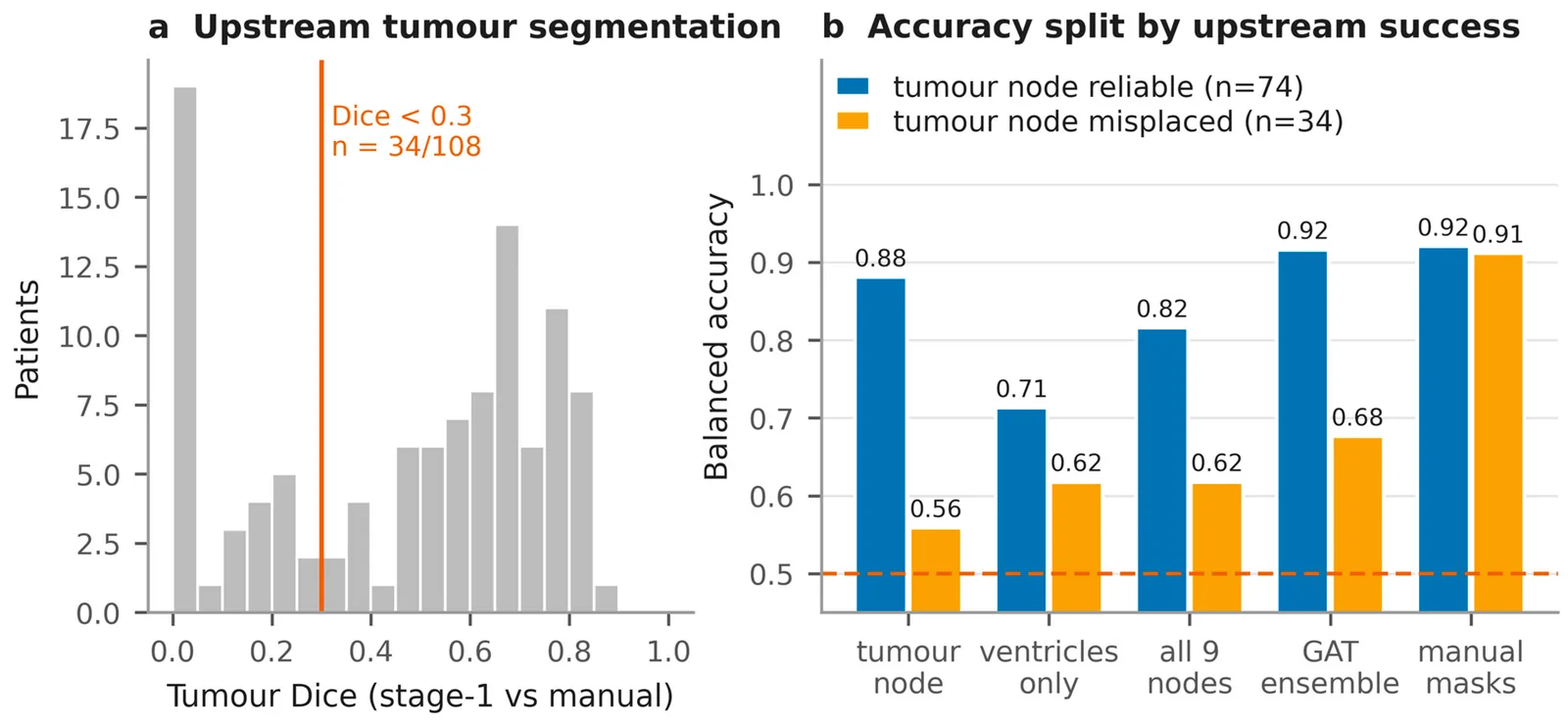
**The automation gap and its dominant failure mode.** (**a**) Distribution of tumour Dice scores across the 108 patients. The dashed line marks the 0.3 threshold used to define the misplaced-tumour group. (**b**) Balanced accuracy for the main input configurations in patients with recovered versus misplaced tumour predictions. Dashed line: chance performance.

Stratifying patients by tumour Dice at a threshold of 0.3 separates 34 patients in whom the tumour node was misplaced (Figure 4b). In that stratum the tumour-node readout falls to near chance while the ventricular nodes still carry signal, and the graph ensemble retains substantially more performance than either.

The character of that stratum is specific: median Dice 0.030, median centroid displacement 40.5 voxels, predicted-to-true volume ratio 0.59, and 13 patients with zero overlap—yet no patient received an empty prediction. The dominant failure pattern was spatial mislocalisation rather than complete absence of a tumour prediction. That a static linear model over all nine nodes does not exploit the surviving ventricular signal, reaching the same 0.618 as the ventricles alone while losing 0.065 in the recovered stratum, is the motivation for a model that can weight nodes per patient.

### 3.5. Robustness to Spatial Mislocalisation

**Figure 5:**
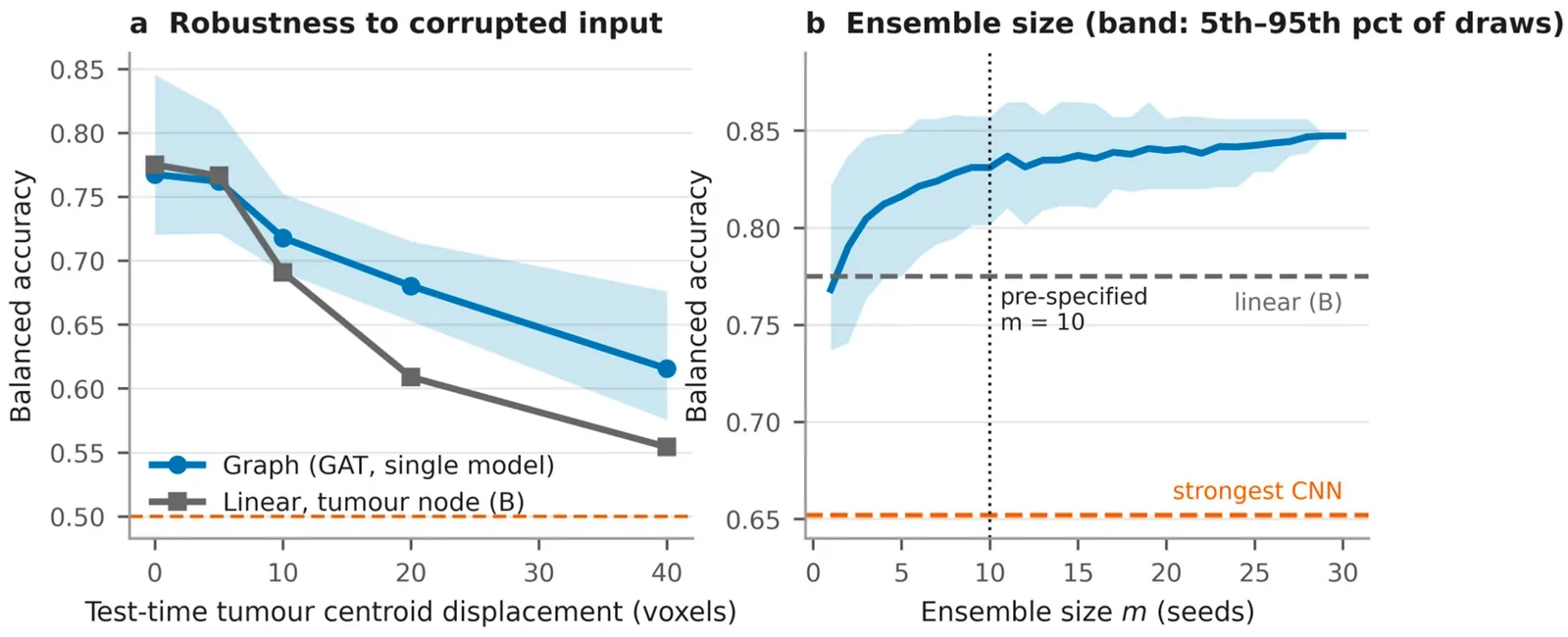
**Robustness to spatial error and sensitivity to ensemble size.** (**a**) Balanced accuracy after controlled displacement of the tumour node at test time; models were trained on unperturbed data, and the graph curve is a single model, not an ensemble, with the band spanning min–max over five seeds. (**b**) Sensitivity to graph ensemble size; line is the mean over 200 random draws of size *m* from a thirty-seed pool, band the 5th–95th percentile. Horizontal references: the linear readout (grey) and the strongest CNN baseline at ten seeds on the same fold partition (orange).

The graph model became more robust than the linear readout as tumour displacement increased (Figure 5a). On clean input the linear readout reached 0.775 against the single graph model’s 0.767 ± 0.042, and at 5 voxels of displacement 0.766 against 0.762 ± 0.035. From 10 voxels onward the ordering reverses: 0.691 against 0.718 ± 0.024 at 10 voxels, 0.609 against 0.680 ± 0.025 at 20, and 0.554 against 0.615 ± 0.040 at 40.

The crossover is consistent across seeds. At 20 and 40 voxels, all five seeds exceed the linear readout, with the worst seed exceeding it by 0.044 and 0.021, respectively, and the crossover was reproduced at the same displacement in an independent execution environment.

This means the graph model’s value lies in tolerance to corrupted geometry rather than in accuracy on clean input. Two observations support that reading. A single graph model (0.768) does not beat the linear readout (0.775), so the graph model’s clean-input advantage is an ensembling effect rather than a per-model one; the gain from ensembling is largest exactly in the misplaced-tumour stratum, where balanced accuracy rises from 0.627 at one seed to 0.735 at thirty. Ensemble size was fixed at ten in advance (Figure 5b); the curve rises steeply to that point (+0.063 from *m* = 1 to *m* = 10) and little beyond it (+0.016 from *m* = 10 to *m* = 30).

### 3.6. Where the Attention Shift Occurs

Testing the hypothesis that the model re-routes attention away from an unreliable tumour node requires separating the two places where attention acts: the message-passing layers, which mix node representations, and the readout, which pools them into a graph-level vector. The results below show that readout attention remained largely unchanged, while attention in the first message-passing layer changed when the tumour prediction was spatially misplaced. This distinction is important, because the readout is the stage that corresponds to the usual interpretability picture of a model “looking elsewhere”, and it is also the stage that is normally inspected.

**Figure 6:**
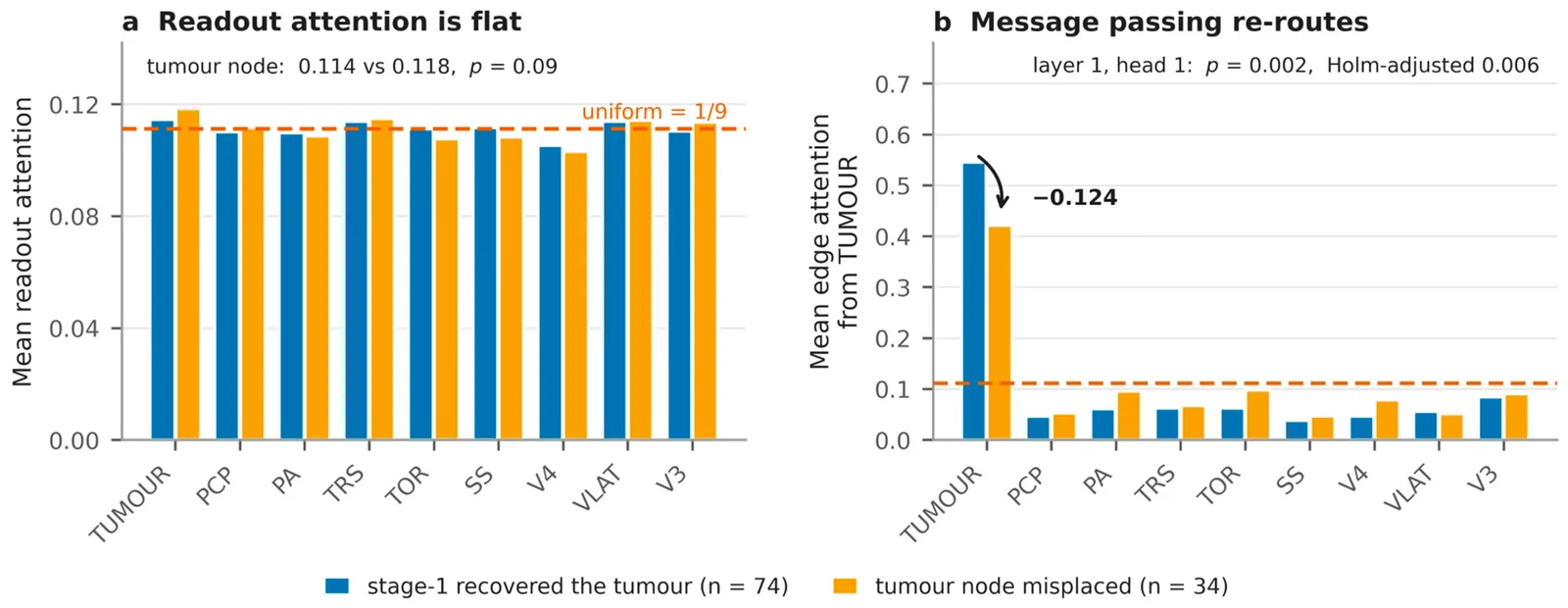
**Where the attention shift occurs.** Mean attention weights over the ten-seed ensemble, split by whether stage-1 recovered the tumour (blue, n = 74) or misplaced it (orange, n = 34). (**a**) Readout attention per node. (**b**) Message-passing attention emitted by the tumour node in the first GAT layer, first head. Dashed line: the uniform value 1/9.

Readout attention was essentially uniform at 1/9 (Figure 6a) and did not differ between segmentation-quality groups: 0.114 against 0.118 for the tumour node (Mann–Whitney *p* = 0.09), 0.114 against 0.114 for the lateral ventricles, 0.105 against 0.103 for the fourth ventricle. This pattern reproduced across CPU and GPU execution, so it is a property of the trained model rather than a setup artefact.

Message-passing attention behaved differently (Figure 6b). The tumour node attends predominantly to itself, with a self-attention coefficient of 0.545 in the first layer against a uniform value of 0.111, and that self-attention fell to 0.421 when stage-1 misplaced the tumour (Table 6). The displaced weight moved to other structures: petrous apex 0.061 → 0.095, torcular 0.062 → 0.098, fourth ventricle 0.046 → 0.079.

The effect is not an artefact of the 0.3 threshold. Treated as a continuous quantity, per-patient tumour self-attention correlates with stage-1 Dice across the whole cohort (Spearman ρ = +0.39, *p* < 0.0001; ρ = +0.43, *p* < 10^−5^ in the first layer alone). It is also localised: both heads of the first message-passing layer survive correction, whereas neither head of the second does. The re-routing therefore happens once, early, where raw node features are first mixed.

This suggests that the model reduces its reliance on the tumour node when that node’s segmentation is unreliable, which is the mechanism Section 3.5 measures behaviourally. The uniformity of the readout does not argue against this mechanism; it indicates that the readout is not the stage at which the change takes place. Because attention was measured rather than experimentally manipulated, this result should be interpreted as evidence of an association rather than proof of causality: establishing causality would require ablating the tumour self-loop and re-measuring robustness under displacement.

## 4. Discussion

The central result of this study is about representation rather than architecture. Holding the cohort, the task and the fold assignment fixed, performance improved as increasingly explicit geometric information was provided to the decision, and the largest single improvement came not from a neural network but from fitting any decision boundary at all to explicit anatomy. The z-profile CNN is the informative intermediate case: it remains a convolutional network, and what it gains over the raw-volume CNN is that the craniocaudal axis is no longer pooled away. The graph representation takes the same step further, since position is provided directly as a node feature.

The parameter-free rule makes the comparison easier to interpret. It shows that, given correct anatomy, this task is close to a sign test. What the learned models add is therefore mainly tolerance to imperfect measurement of that anatomy, rather than a better description of the task itself.

These findings do not show that graph neural networks are generally superior to convolutional networks for paediatric brain MRI. The comparison instead suggests that the representation of anatomical information is important for this task: a simple geometric representation already accounted for a large part of the class separation, and the GAT was more robust when the tumour location was spatially displaced. On clean predicted input, a single graph model did not outperform a five-feature logistic regression. Its advantages appeared in two specific situations—when predictions were averaged across seeds, and when the input was degraded—and both are relevant clinically, because a deployed segmenter will not be perfect and its errors are not uniform across structures.

Where the automated pipeline loses accuracy is upstream. The gap between manual and predicted masks was associated primarily with spatial mislocalisation of the tumour rather than with failure to produce a tumour prediction at all, which identifies spatial fidelity in segmentation, not classifier capacity, as the target for future work. Segmentation quality also trends with structure size across the eight non-tumour nodes (Spearman ρ = 0.45), but with only eight structures this is not significant (*p* = 0.26) and is stated as a trend rather than a finding.

Part of the residual error is a property of the cohort rather than of any particular model. The errors that remain under manual annotation are concentrated in lesions centred within a few voxels of the tentorial plane, and they do not disappear when the classifier is given the whole cohort to fit, which locates the limitation in what a tumour centroid can express rather than in the amount of training data available to the decision layer. A single point summarises a lesion whose compartment is determined by the position of its bulk relative to a curved membrane, and for a small lesion centred at the tentorial incisura the two need not agree. This sets a practical bound on the manual-mask configuration that is independent of segmentation quality, and it is the reason that configuration is presented as a reference for this decision layer rather than as a ceiling on the task.

Several further properties of the cohort bear on how these results should be read. The task definition itself removes the hardest cases: of 141 annotated patients, 16 carried a bitentorial label and were excluded, so the binary problem studied here is easier than the clinical question it abstracts. The two data sources are also unequal, with 91 local patients of whom 44% are infratentorial, compared with 17 externally sourced patients of whom 65% are, so the external subset is too small to support a separate estimate and source cannot be separated from class at this cohort size. The structures that define the tentorial surface are in addition the hardest to segment: the torcular and the petrous apex reached median Dice scores of 0.267 and 0.316, with 41% and 24% of patients below 0.1, so the reference surface itself is estimated from the least reliable part of the annotation. Finally, the resampling to 256 × 256 × 32 compresses the craniocaudal axis and removes most of the tentorial tilt; Section 3.2 shows that this costs little once a threshold is fitted, but it means that the curvature which distinguishes the tentorium from a plane is largely absent from the representation the models receive.

The attention analysis carries a methodological lesson beyond this dataset. The interpretable story—attention routing around an unreliable node—turns out to be correct, but only at one of the two stages where attention acts, and the stage that is conventionally inspected is the one where it is absent. Had the analysis stopped at the readout, as such analyses usually do, the conclusion would have been that no re-routing occurs. Reporting attention from a single stage of a multi-stage architecture can therefore support either conclusion, which makes it important to state explicitly which stage was measured.

The accuracies reported here are low relative to the 95–100% common in this literature. That difference is best read as a statement about evaluation protocol rather than about model quality: the splits are patient-level and subject-grouped, the predictions are out-of-fold at both stages, the comparisons are paired on verified-identical folds, and every estimate carries an interval.

### Limitations and Future Directions

Four limitations bound the conclusions of this study.

First, the cohort is small and single-study. The 108 patients come from two data sources that differ in class balance and acquisition protocol, so source is a potential confounder that a cohort of this size cannot resolve, and no independent external test set was available. The sample size also limits the precision of comparisons between the graph models: the difference between the GAT and the linear readout (+0.064) has a confidence interval that touches zero, and the same computation under a different hardware and library stack returned [−0.028, +0.120], so numerical non-determinism alone moves this interval across zero. Whether any externally sourced case also appears in public releases used to train previously published models has not been formally checked, and a reader intending to compare against such a model should treat overlap as unexcluded.

Second, the automation gap depends on the upstream segmentation model, which was not retrained for this study. The measured gap should therefore be interpreted as specific to this segmentation pipeline rather than as a general estimate of the cost of automation, and the manual-mask configuration is a bound on this pipeline rather than a deployable configuration.

Third, the mechanistic interpretation remains partly correlational. The attention analysis provides evidence that message-passing attention changes when tumour segmentation is unreliable, but it does not establish causality. Attention ablation or intervention experiments would be required to determine whether this shift directly causes the observed robustness.

Fourth, each case was annotated once. No second reader was available, so inter-rater variability in the landmark annotations could not be quantified, and the manual-mask configuration inherits whatever systematic bias that single reading carries. The reference label was recorded independently of the annotation, which protects the comparison from circularity, but it too rests on the judgement of one clinician. A multi-reader subset would allow both sources of variability to be bounded.

These observations indicate what an extension of this work would require. Increasing the number of patients would help less than increasing the number of the right patients: because the residual errors are concentrated in lesions centred close to the tentorial plane, a larger cohort enriched for near-boundary and small lesions would constrain the decision boundary where it is actually uncertain, whereas additional unambiguous cases would largely confirm what the present cohort already establishes. A larger and more balanced cohort would also allow the bitentorial cases to be reinstated as a third class rather than excluded, bringing the task closer to the clinical question, and would make it possible to hold out an independent multi-centre test set instead of relying on cross-validation alone. Two further extensions follow directly from the error analysis. Repeat annotation of the borderline cases by more than one reader would establish how much of the residual disagreement is intrinsic to the label, and would support treating compartment as a graded quantity—the signed distance to the tentorial surface—rather than as a binary one. Annotation effort directed specifically at the small tentorial structures, which are both the least reliably segmented and the ones that define the reference surface, would be expected to reduce the automation gap more efficiently than further work on the classifier. Acquiring or retaining finer through-plane resolution would allow the same pipeline to be evaluated where the tentorial curvature is preserved, and would test whether that curvature carries information the present representation cannot convey.

## 5. Conclusions

This study examined the role of the anatomical representation in paediatric supratentorial/infratentorial tumour localisation. Under the same cohort and the same patient-level evaluation protocol, balanced accuracy increased as more explicit geometric information was provided to the decision. A simple geometric representation already accounted for a large part of the class separation, indicating that the relevant information is low-dimensional and can be computed directly from the anatomy instead of being learned from raw voxels.

The main limitation in the automated pipeline was upstream segmentation. The performance gap between manual and predicted masks was associated primarily with spatial mislocalisation of the tumour rather than with complete failure to produce a tumour prediction. This identifies segmentation accuracy and spatial fidelity as important targets for future work. Separating the two stages at which attention acts showed that the re-routing behind the graph model’s tolerance of such errors occurs in the first message-passing layer and not at the readout, which is the stage these analyses conventionally inspect.

The graph attention model was more robust when the tumour location was spatially displaced. With the present sample size, however, the study does not provide sufficient evidence that it performs better than the linear graph readout when the input is correctly localised. Overall, for this geometrically defined task and at this cohort size, representing anatomical structure explicitly was more effective than asking a high-capacity network to learn the same geometry from voxels.

## Figures and Tables

**Table 1:** The model and input configurations compared in this study.

Configuration	Input	Model
D	raw MRI volume	3D CNN, three variants
A	**manual** masks → node features	logistic regression on the tumour node (reference)
B	**predicted** masks → tumour node (5 numbers)	logistic regression
B′	predicted masks → all 9 nodes (45 numbers)	logistic regression
C	predicted masks → 9-node graph	graph attention network

**Table 2:** Simple readouts of the anatomy, by mask source (balanced accuracy; 95% CI where computed).

Decision Procedure	Manual Masks	Predicted Masks
Tentorial surface, sign only (no fitted parameters)	0.777 [0.704, 0.849]	0.681 [0.608, 0.759]
Signed distance to the surface, one fitted threshold	0.874 [0.813, 0.932]	0.787 [0.704, 0.861]
Tumour *z* versus mean landmark *z* (no fitted parameters)	0.769 [0.692, 0.842]	0.671 [0.593, 0.746]
Tumour *z* coordinate alone, fitted	0.856	0.759
Tumour *x*, *y*, *z* + log volume + presence (configurations A and B)	0.918	0.775
Full nine-node graph + GAT (configuration C)	—	0.838

**Table 3:** Paired comparisons against the CNN baselines.

Baseline (Ten-Seed Ensemble, Identical Folds)	vs. B—Linear Readout (0.775)	vs. C—GAT (0.838)
3D CNN, raw volume (scratch3d)—0.522	+0.253 [0.135, 0.367], *p* = 0.0001	+0.317 [0.210, 0.423], *p* < 0.0001
3D CNN + coordinate channel (scratch3d_coord)—0.538	+0.237 [0.117, 0.350], *p* = 0.0003	+0.300 [0.195, 0.405], *p* < 0.0001
3D CNN + craniocaudal profile (scratch3d_zprofile)—0.652	+0.123 [0.000, 0.247], *p* = 0.073	+0.187 [0.072, 0.300], *p* = 0.0029
B—graph, linear readout, tumour node—0.775	—	+0.064 [0.000, 0.130], *p* = 0.092

**Table 4:** Class-wise out-of-fold performance of the graph configurations.

Configuration	SUPRA Correct	INFRA Correct	SUPRA Rate	INFRA Rate	Balanced Accuracy
A—manual masks (reference)	51/57	48/51	0.895	0.941	0.918
B—predicted masks, linear, tumour node	47/57	37/51	0.825	0.725	0.775
B′—predicted masks, linear, nine nodes	43/57	38/51	0.754	0.745	0.750
C—predicted masks, GAT, ten-seed ensemble	52/57	39/51	0.912	0.765	0.838

**Table 5:** Stage-1 segmentation quality by structure (n = patients in which the structure was annotated).

Node	n	Dice (Median)	Dice < 0.1	Centroid Error (Voxels, Median)
VLAT	108	0.722	1%	3.3
V4	106	0.532	12%	2.4
V3	106	0.517	10%	3.5
PCP	105	0.516	4%	3.6
TUMOUR	108	0.561	19%	9.8
SS	103	0.416	18%	13.1
TRS	107	0.350	10%	13.6
PA	102	0.316	24%	14.3
TOR	106	0.267	41%	6.1

**Table 6:** Tumour self-attention by stage-1 segmentation quality, ten-seed mean.

Attention Stage	Tumour Recovered (n = 74)	Tumour Misplaced (n = 34)	Mann–Whitney *p*	Holm-Adjusted *p*
Message passing, layer 1, head 1	0.545	0.421	0.002	0.006
Message passing, layer 1, head 2	0.494	0.389	0.004	0.013
Message passing, layer 2, head 1	0.434	0.412	0.187	0.187
Message passing, layer 2, head 2	0.436	0.397	0.063	0.125
Pooled over layers and heads	0.477	0.405	0.004	—
Readout (for comparison)	0.114	0.118	0.091	—

## Data Availability

The underlying imaging data cannot be shared directly; requests are handled through the Children’s Brain Tumor Network data use agreement and through the institutional ethics approval covering the local archive. The analysis code—preprocessing, graph construction, model training, and the evaluation and statistical procedures—together with the de-identified derived node table that reproduces every quantitative result in Section 3 (centroids, volumes, presence flags, class labels, fold assignment and per-patient tumour Dice, containing no patient identifiers and no imaging), is available from the author on reasonable request. Because StratifiedGroupKFold assignments depend on the scikit-learn version, reproduction requires the software environment reported in Section 2.7.
